# An exploration of conditions proposed to trigger the Ebola virus glycoprotein for fusion

**DOI:** 10.1371/journal.pone.0219312

**Published:** 2019-07-05

**Authors:** Lucie Fénéant, Katarzyna M. Szymańska-de Wijs, Elizabeth A. Nelson, Judith M. White

**Affiliations:** 1 Department of Cell Biology, University of Virginia, Charlottesville, Virginia, United States of America; 2 Department of Microbiology, University of Virginia, Charlottesville, Virginia, United States of America; Deutsches Primatenzentrum GmbH - Leibniz-Institut fur Primatenforschung, GERMANY

## Abstract

Ebolaviruses continue to inflict horrific disease and instill fear. The 2013–2016 outbreak in Western Africa caused unfathomable morbidity and mortality (over 11,000 deaths), and the second largest outbreak is on-going in the Democratic Republic of the Congo. The first stage of an Ebolavirus infection is entry, culminating in delivery of the viral genome into the cytoplasm to initiate replication. Among enveloped viruses, Ebolaviruses use a complex entry pathway: they bind to attachment factors on cell surfaces, are engulfed by macropinocytosis, and traffic through the endosomal system. *En route*, the receptor binding subunit of the glycoprotein (GP) is reduced from ~130 to ~19 kDa by cathepsins. This event allows cleaved GP (GP_cl_) to bind to Niemann-Pick C1 (NPC1), its endosomal receptor. The virus then fuses with a late endosomal membrane, but how this occurs remains a subject of debate. An early, but standing, observation is that entry of particles bearing GP_cl_ is inhibited by agents that raise endosomal pH or inhibit cysteine proteases, suggesting the need for an additional factor(s). Yet, some have concluded that NPC1 is sufficient to trigger the fusion activity of GP_cl_. Here, we re-examined this question using sensitive cell-cell and pseudovirus-cell fusion assays. We did not observe detectable GP_cl_-mediated fusion with NPC1 or its GP_cl_ binding domain at any pH tested, while robust fusion was consistently observed with GP from lymphocytic choriomeningitis virus at low pH. Addition of proposed fusion-enhancing factors—cations (Ca^++^ and K^+^), a reducing agent, the anionic lipid Bis(Monoacylglycero)Phosphate, and a mixture of cathepsins B and L—did not induce detectable fusion. Our findings are in line with the earlier proposal that an additional factor is required to trigger the full fusion activity of GP_cl_ after binding to NPC1. We discuss caveats to our study and what the missing factor(s) might be.

## Introduction

Ebolaviruses are single-stranded negative-sense enveloped viruses belonging to the *Filoviridae* family. Five species are known with four causing hemorrhagic fevers in humans, including Ebola virus (EBOV), the species responsible for the deadliest epidemic [1,2]. With over 11,000 deaths during the 2013–2016 outbreak in Western Africa and no antiviral drug approved, understanding the biology of this virus is essential to develop specific treatments.

EBOV enters host cells by binding to attachment factors such as lectins and TIM/TAM family members through interactions, respectively, with the viral glycoprotein (GP) and phospholipids in the viral envelope [3]. GP is a class I fusion protein present at the surface of the virion as a trimer of heterodimers, each comprised of two subunits linked by a disulfide bond: the highly glycosylated receptor-binding subunit (GP1) and the fusion subunit (GP2). After internalization through a macropinocytotic pathway [3,4], GP1 is cleaved at low pH by endosomal cysteine-proteases, namely cathepsins B and L, removing the mucin domain and glycan cap and producing a cleaved form of GP1 (~19 kDa) [5–7]. Then GP1 binds to its intracellular receptor, Niemann-Pick C1 (NPC1), a cholesterol transporter located in late endosomes/lysosomes [8–10]. The utilization of intracellular viral receptors is a fairly new concept in virology, with all known filoviruses employing NPC1 and the arenavirus, Lassa virus (LASV), using Lamp1 [2,11–15]. While Lamp1 is thought to raise the pH-threshold for fusion [13,14], the exact role of NPC1 in EBOV entry is yet to be determined. It is postulated that after binding to NPC1, cathepsin-cleaved GP (GP_cl_) undergoes further conformational changes empowering GP2 to mediate fusion [16].

The factors needed to trigger the fusion activity of GP_cl_ remain under debate. Early work showed that host cell entry mediated by GP_cl_ remains sensitive to lysosomotropic agents and the cysteine protease inhibitor E64d [6]. These findings suggested that GP_cl_ requires low pH and an additional activity inhibited by E64d, perhaps additional cathepsin cleavage, to trigger its fusion ability. Additional cell biological [17–20] and biochemical studies [21–24] supported this concept. Furthermore, findings stated in [10] suggested that low pH and engagement of the C-loop of NPC1, the binding domain for GP_cl_ within NPC1, are not sufficient to trigger fusion. The endosomal two pore calcium channel 2 (TPC2) was subsequently identified as a late entry factor for EBOV [25], but its exact role in fusion is not known. In contrast to the literature cited above suggesting needs for low pH, NPC1, and an additional factor(s) to support GP_cl_-mediated fusion, Markosyan et al. [26] proposed that binding to NPC1 is sufficient to induce GP_cl_ to induce fusion, even at neutral pH.

In the present study, we revisited the question of how the cleaved form of EBOV GP (GP_cl)_ is triggered for fusion by testing the roles of NPC1, low pH, and other potential triggering factors. The assays employed included highly sensitive luciferase-based cell-cell and pseudovirus-cell fusion assays, as well as a flow cytometric-based assay for cell-cell hemifusion. Our findings suggest that neither the NPC1-C-loop nor full-length NPC1 are sufficient to trigger significant fusion at any pH tested. Even the presence of additional factors including a reducing agent, a negatively charged lipid abundant in late endosomes/lysosomes, cathepsins, or the cations Ca^++^ and K^+^ did not elicit detectable fusion. Taken together, our results suggest that cathepsin primed EBOV-GP requires one or more factors in addition to NPC1 and low pH to mediate a fusion reaction sufficiently robust to allow entry of the EBOV genome into the cytoplasm to initiate replication.

## Materials and methods

### Chemicals, enzymes, cell cultures and media

Dulbecco’s modified Eagle’s medium (DMEM), phenol-red free DMEM, trypsin-EDTA 0.05%, phenol-red free trypsin-EDTA 0.5%, Hank’s Balanced Salt Solution (HBSS), sodium pyruvate, antibiotic/antimycotic, EZ-Link Sulfo-NHS-LC-Biotin, DiI, and DiO were from ThermoFisher Scientific. Phosphate-buffered saline (PBS) was from Corning. Cosmic calf serum (CCS), Supplemented calf serum (SCS) and Fetal Bovine Serum (FBS) were from HyClone. Fibronectin was from Millipore. Polyethylenimine (PEI*)* and Non-Enzymatic Cell Dissociation Solution were from Sigma. Thermolysin was from VitaCyte. Lipofectamine 2000 was from Invitrogen and zeocin was from Invivogen. Cathepsin B was from Athens Research and Technology. Cathepsin L was a gift from Dusan Turk. Cathepsin B and L substrates (Z-Arg-Arg-7-AMC and Z-Phe-Arg-7-AMC, respectively) were from Calbiochem. Bis(monoacylglycero)phosphate S,R isomer (BMP) was from Avanti Polar Lipids. Delipidated BSA was a gift from David Castle (University of Virginia). EnduRen and the Dual-Glo Luciferase Assay System were from Promega.

HEK293T/17 cells were obtained from ATCC via the University of Virginia Tissue Culture Facility. COS7 cells were a kind gift from Douglas DeSimone at the University of Virginia. BHK21 were a kind gift from James Casanova at the University of Virginia. HEK293T/17 cells were maintained in DMEM containing 10% CCS. COS7 cells were maintained in DMEM containing 10% FBS, 1% sodium pyruvate and 1% antibiotic/antimycotic. BHK21 cells were maintained in DMEM containing 10% SCS.

Stable clones expressing full length NPC1 at the cell surface were generated as follows. The pCMV6-XL4-hNPC1 plasmid was from OriGene. DNA encoding full length NPC1 was excised from this plasmid using the enzymes NheI and XhoI and cloned into the vector pCDNA3.1Zeo(+) (ThermoFisher Scientific) cut with the same enzymes using standard procedures. 500,000 HEK293T/17 cells were seeded in 6-well plates. The day after, they were transfected with 1μg of pCDNA3.1Zeo(+)-NPC1, empty vector (for negative control cell lines), or not transfected (to assess cell viability following zeocin exposure) using Lipofectamine 2000, according to the manufacturer instructions. Two days after transfection, the cells were replated in a 10cm^2^ dish and incubated with 500 μg/ml of zeocin in complete DMEM. Cells were passaged twice in 500 μg/ml zeocin, and surviving cells were then maintained in complete DMEM + 500 μg/ml zeocin until they formed colonies. Colonies were individually picked and transferred to wells of a 24 well plate. The cells were then expanded and characterized for NPC1 expression at the plasma membrane by cell surface biotinylation.

### Viruses and plasmids

The EBOV-FL-GP-V5 and EBOV-21K-GP-V5 plasmids (Mayinga strain) were kind gifts from Paul Bates at the University of Pennsylvania. The pFurin plasmid was a kind gift from Gary Thomas at the University of Pittsburgh. The Lymphocytic Choriomeningitis Virus (LCMV)-GP plasmid was a kind gift from Juan de la Torre at the Scripps Research Institute, La Jolla, CA. The VSV-G plasmid was a kind gift from Michael Whitt at the University of Tennessee. Plasmids encoding soluble and membrane bound NPC1-C-loop (in pDisplay vectors) were prepared by James Simmons according to published procedures [10,27]. The pcDNA3-luciferase (Firefly) plasmid was from Addgene. DSP1-7 and DSP8-11 (Dual Split Plasmids) were a kind gift from Naoyuki Kondo [28].

### Antibodies and immunoprecipitation reagents

The monoclonal mouse-α-GP1 H3C8 antibody was a kind gift from Carolyn Wilson of the Food and Drug Administration. Rat-α-HA-HRP 3F10, rabbit α-GAPDH and mouse-α-beta tubulin were from Sigma Aldrich. The monoclonal rabbit-α-NPC1 (ab134113) was from Abcam. The anti-beta tubulin mouse monoclonal antibody was from Sigma Aldrich. The sheep-α-mouse-HRP, the donkey-α-rabbit-HRP and the streptavidin magnetic sepharose beads were from GE Healthcare. The α-rabbit IR800, α-mouse IR680, and α-rat IR680 antibodies were from Licor. The mouse α-VSV-M antibody (23H2) was a gift from Michael Whitt.

### In Cell Westerns

In Cell Westerns were performed essentially as described [13,29]. COS7 cells expressing EBOV GP_CL_ (21 kDa) or NPC1-C-Loop and VSV pseudoviruses were prepared as described below. Cells were then seeded on fibronectin coated wells (15,000 cells/well, 96 well plates) and grown overnight. For NPC1 C-loop binding to EBOV GP_CL_ cells, 10μg/mL soluble NPC1 C-loop prepared as described [10, 27] was added per well. For VSV pseudovirus binding, 10 μg of pseudoviruses were added per well. The cells were then centrifuged (1h, 4°C), transferred to ice, washed once (cold PBS), fixed (2% paraformaldehyde, 15 min, RT), permeabilized (5 washes, 0.1% Trition X-100 in PBS, 5 min per wash), and blocked (1.5 h, RT, Licor Odyssey Blocking Buffer). Primary antibodies (α-GAPDH, α-VSV-M (23H2) or α- HA 3F10, in blocking buffer) were added and the cells incubated overnight at 4°C. After washing (3 times, PBS plus 0.1% Tween-20, 5 min per wash), secondary antibodies (α-rabbit IR800, α-mouse IR680 or α-rat IR680, in blocking buffer with 0.2% Tween-20) were added, the plates incubated 1 h at RT and then washed 4 times in PBS with 0.1% Tween-20 and once with PBS (5 min/wash). After drying, the plates were scanned (Licor Odyssey CLx near-infrared imaging system). Red and green intensities (per well) were determined (“Measure” function, ImageJ), and their ratios (HA to GAPDH or VSV-M to GAPDH) calculated and averaged.

### Cell-cell fusion assay

This assay was performed as previously described [13]. Briefly, HEK293T/17 were used to generate, by transfection, both effector cells, expressing EBOV-21K-GP, LCMV-GP, or no viral glycoprotein (No-GP) as well as target cells, expressing or not, NPC1-C-loop or full-length NPC1. An additional 0.5 μg/well of a plasmid encoding furin was transfected alongside the EBOV-GP plasmid to generate the 21K form at the cell surface. Two days after transfection, effector cells were loaded with EnduRen before being cocultured with target cells for 3h. Then, a pH pulse was applied for 5 min at 37°C (unless otherwise stated) in HMS fusion buffer (100 mM NaCl, 15 mM HEPES, 15 mM succinate, 15 mM MES, 2mg/mL glucose) adjusted to the appropriate pH (with additional components, as indicated). The medium was then replaced, and luciferase activity was measured 1 h after the pH pulses.

For cell-cell fusion assays assessing the effect of exogenous cathepsins B and L, cathepsins were pre-activated in preactivation buffer (100mM sodium acetate, 1mM EDTA, pH 4.5 containing fresh 5mM DTT) for 15 min at 37°C. The preactivated cathepsins were then diluted to 5μg/ml in HMS fusion buffer at the indicated pH and incubated at RT for 15 min. Preactivated cathepsins in HMS fusion buffer at the indicated pH were then used immediately, i.e. immediately added to cells for 5 min at 37°C. After that, the pH was reneutralized and the cells were processed as described above for the general cell-cell fusion assay. To assess cathepsin activity, the preactivated cathepsins were diluted to 5μg/ml in HMS fusion buffer, pH 5.0 with or without 10μM E64 (a cysteine protease inhibitor) or 1μM CA074 (a cathepsin B inhibitor) and were incubated at RT for 15 min. Cathepsin B and L substrates (100 μM in ddH_2_O) were added in a 1:1 ratio to the preactivated cathepsins, and the mixtures were incubated for 5 min at 37°C. Fluorescence (ex/em: 360/460) was measured using a BioTek Synergy HT plate reader.

For cell-cell fusion assays assessing the effect of adding BMP to the plasma membrane, cocultured cells were chilled at 10°C for 15 min and then washed once with cold PBS. Next, a mixture of 5 μM BMP and 5 μM delipidated BSA was added to the cells in serum free DMEM for 5 min at 10°C. Immediately after, the cells were rinsed once with PBS and subjected to a pH pulse. The cells were then processed as above for the general cell-cell fusion assay.

### Lipid mixing (hemifusion) assay

HEK293T/17 cells were seeded in 6-well plates (6.75 x 10^5^ cells/well). The next day, to generate effector cells expressing EBOV-21K-GP, LCMV- GP, or no viral glycoprotein (No GP), cells were transfected with 1 μg/well of glycoprotein plasmids. An additional 0.5 μg/well of furin plasmid was transfected alongside the EBOV-GP plasmid to generate the 21K form at the cell surface. To generate target cells, expressing or not expressing NPC1-C-loop, cells were transfected with 1 μg/well of membrane-anchored NPC1-C-loop plasmid. Cells were transfected using Lipofectamine 2000, according to the manufacturer’s instructions. Twenty-four hours post-transfection, effector and target cells were stained with 10 μM DiO and 2 μM DiI, respectively, for 30 min in complete DMEM at 37°C. Cells were rinsed with PBS and lifted with Non-Enzymatic Cell-Dissociation Solution. Cells were pelleted and resuspended in complete phenol-red free DMEM and 160,000 effector cells were cocultured with 40,000 target cells in a fibronectin coated 96-well plate for 1 h at 37°C. Then, a pH pulse was applied, in HMS fusion buffer adjusted to the appropriate pH, for 5 min at 37°C. pH was re-neutralized by replacing the buffer with complete DMEM and cells were returned to 37°C. After 2 h, cells were lifted with phenol red-free trypsin, fixed in 4% PFA and analyzed on a BD FACSCalibur. Data were analyzed with FlowJo and plotted using FCS Express.

### VSV pseudoviruses production

The protocol to produce VSV pseudoviruses encoding *Renilla* luciferase was described previously [13]. Briefly, BHK21 cells in 10 cm dishes were transfected with plasmid encoding EBOV-FL-GP, EBOV-21K-GP or LCMV-GP. An additional 6 μg of plasmid encoding furin was transfected alongside cells transfected with EBOV-21K-GP, to allow for expression of the 21K cleaved form of GP on the surface of the particles. The next day, cells were infected with VSV-ΔG helper virus encoding *Renilla* luciferase. Supernatants containing pseudoviruses were then harvested, pelleted through a 20% sucrose cushion, and resuspended in a sterile solution of 10% sucrose in HM buffer (130 mM NaCl, 20 mM HEPES, 20 mM MES, pH 7.4).

### Thermolysin cleavage

Twenty-five micrograms of EBOV-FL-GP VSV pseudoviruses were cleaved to the 19K form of GP with 0.25 mg/ml freshly prepared thermolysin in the presence of 2 mM of CaCl_2_. Viruses were incubated for 30 min at 37°C and the reaction was quenched by adding 500 μM phosphoramidon.

### Forced fusion at the plasma membrane assay

This assay was described previously in [13]. Briefly, COS7 cells were transfected to express membrane-anchored NPC1-C-loop and Firefly luciferase. After two days, the cells were chilled on ice for 15 min and incubated with EBOV-21K-GP, EBOV-19K-GP, or LCMV-GP VSV-luciferase (*Renilla*) pseudoviruses (at least 3 wells per sample). After binding in the cold, a pH pulse was applied for 5 min at 37°C in HMS buffer adjusted to the indicated pH value. The buffer was then replaced with DMEM containing 40 mM NH_4_Cl. Twenty-four hours later, luciferase activities were measured using the Dual-Glo Luciferase Assay System (Promega). Viral fusion with the plasma membrane was assessed as the ratio of *Renilla* luciferase activity (an indicator for virus replication) over Firefly luciferase activity (to account for the number of cells).

### Cell-surface biotinylation

Cells to be analyzed by cell-surface biotinylation were seeded on a 6-well plate. Cells were chilled on ice for 15 min and rinsed three times with cold HBSS. Cells were then incubated for 30 min on ice with 0.2 mg/ml of EZ-Link Sulfo-NHS-LC-Biotin diluted in HBSS. Cells were then washed three time with PBS 0.6% BSA, 100 mM glycine, pH 7 and lysed in PBS-1% Triton X-100. Lysates were precipitated using streptavidin magnetic sepharose beads according to the manufacturer’s instructions. The beads were boiled in SDS-PAGE sample buffer, collected with a magnet, and the eluted fraction was analyzed by blotting SDS-PAGE gels for the indicated proteins.

### Biosafety

All work with VSV pseudoviruses (encoding LCMV and EBOV GPs) and primate cells was performed at the Biosafety 2 level, in accordance with its review and approval by the Institutional Biosafety Committee of the University of Virginia School of Medicine.

## Results

### NPC1 C-loop and low pH are not sufficient to trigger detectable fusion by GP_cl_

NPC1 is the intracellular receptor for EBOV [8,9], and its luminally-oriented C-loop is sufficient to bind cathepsin-primed EBOV-GP. Sufficiency of the C-loop for binding cleaved EBOV GP (GP_cl_) and for mediating entry through endosomes was demonstrated using NPC1 domain constructs, deletion mutants and chimeras between NPC1 and Niemann-Pick C1 Like 1 (NPC1L1), a NPC1 paralog [10,30]. But the latter studies did not address the sufficiency of the C-loop for fusion *per se*. We therefore first asked whether NPC1 C-Loop and low pH are sufficient to drive the fusion activity of GP_cl_.

**Fig 1:**
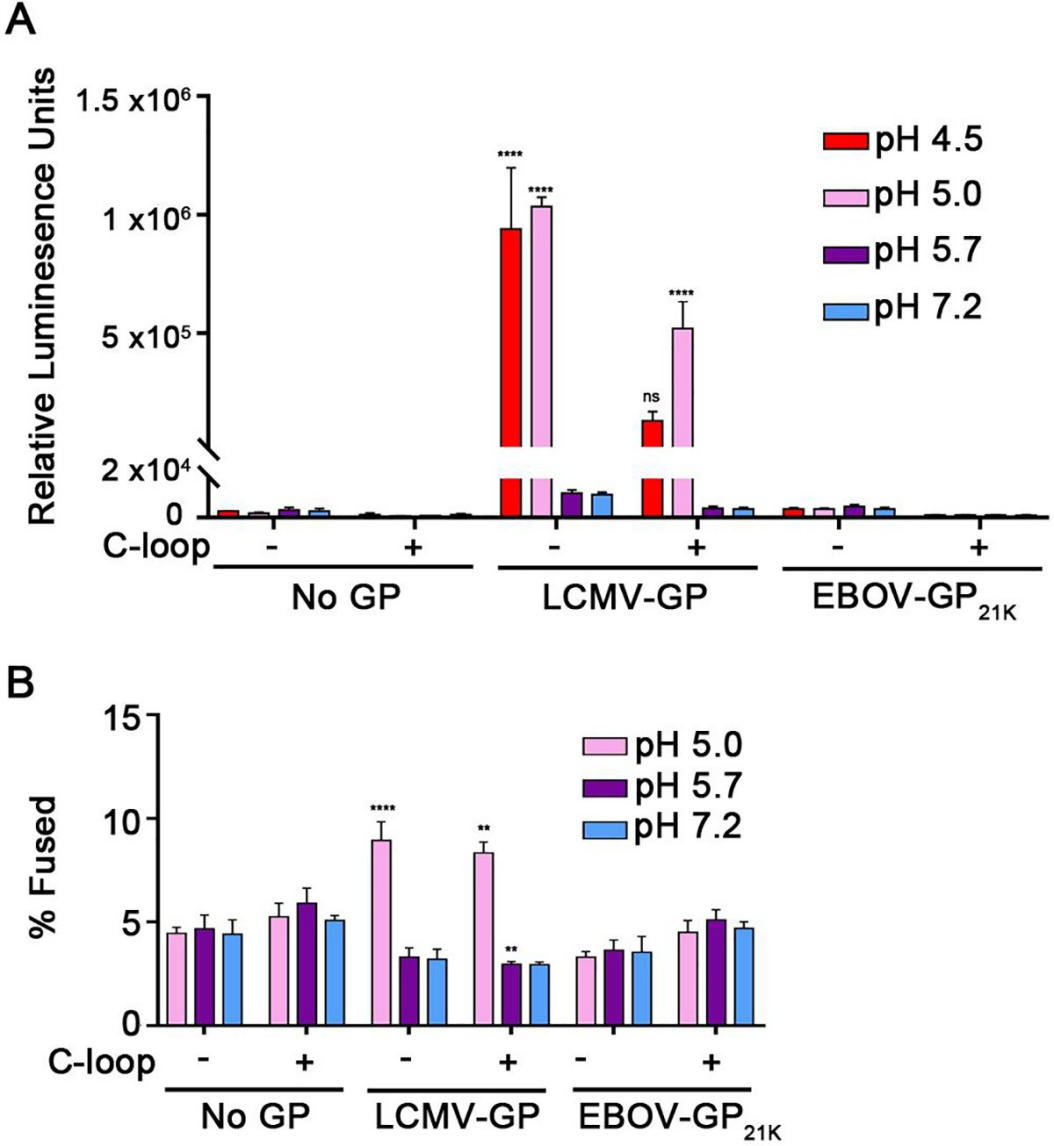
NPC1-C-loop and low pH and are not sufficient to trigger detectable cell-cell fusion (CCF) activity of EBOV-GP_cl_. **(A) Cell-cell fusion: content mixing:** Effector HEK293T/17 cells were transfected to express LCMV-GP, EBOV-21K-GP or No GP and DSP_1-7_. Target HEK293T/17 cells were transfected to express cell surface directed NPC1-C-loop and DSP_8-11_. Effector and target cells were cocultured for 3 h, subjected to a 5-min pulse at the indicated pH at 37°C, and returned to the 37°C incubator. After 1 h, luciferase activity was measured as described in the Methods section. **(B) Cell-cell fusion: lipid mixing:** Effector HEK293T/17 cells expressing No or the indicated GP were labeled with DiO. Target HEK293T/17 cells were transfected to express surface-directed NPC1-C-loop and labeled with DiI. Effector and target cells were then cocultured for 1 h at 37°C, subjected to a 5-min pulse at the indicated pH (at 37°C), and then returned to the 37°C incubator. After 2 h, the cells were fixed and analyzed by flow cytometry. The percentage of double positive (DiI and DiO) cells eliciting FRET (fused and hemifused) was calculated from the total number of stained cells, as described in the Methods section (see **S3 Fig** for sample FACS plots). Results in **A** represent mean +/- SD of triplicate samples from one of two experiments with similar results. Results in **B** represent mean +/- SEM of data from three independent experiments, each performed with triplicate samples. Statistical analyses are based on two-way ANOVA tests: for **A** each sample was compared to the respective No GP sample and for **B** compared to the pH 7.2 value in each group. ****p < 0.0001; **p < 0.01.

We first explored the sufficiency of NPC1 C-Loop and low pH using a highly sensitive cell-cell fusion (CCF) assay. For this assay, effector cells expressing GP_cl_ (**S1A Fig**) were cocultured with target cells expressing NPC1 C-loop at the cell surface (**S1B Fig)**. For effector cells, to avoid the toxicity of thermolysin treatment of cells expressing full-length EBOV-GP (to generate 19 kDa GP), we co-expressed DNAs encoding EBOV GP with a furin cleavage site at positions 203–206 and furin, yielding 21 kDa GP, a form capable of binding the C-Loop [18,26,31] (also see **S2A Fig)**. Effector and target cells each additionally expressed one half of a split luciferase/split GFP construct (DSP_1-7_ and DSP_8-11_, respectively). If fusion occurs, the resulting syncytia express luciferase and GFP [28]. Effector cells expressing GP from Lymphocytic Choriomeningitis Virus (LCMV) were used as a positive control. As seen in **Fig 1** (panel A), after a pulse at pH 4.5, 5.0, 5.7, or 7.2, no cell-cell fusion (content mixing) was observed for cells expressing EBOV-21K-GP, with target cells that either did or did not express C-loop at their surface; the observed luciferase activity levels were comparable to those seen with effector cells harboring no GP. In contrast, robust luciferase signals indicative of CCF were observed for cells expressing LCMV-GP at pH 4.5 and 5.0 with target cells that did or did not express NPC1 C-loop (**Fig 1A**), consistent with the pH requirements for LCMV GP-mediated fusion [13,32]. This suggested that NPC1-C-loop and low pH are not sufficient to trigger EBOV-GP_cl_ to mediate CCF leading to content mixing.

**Fig 2:**
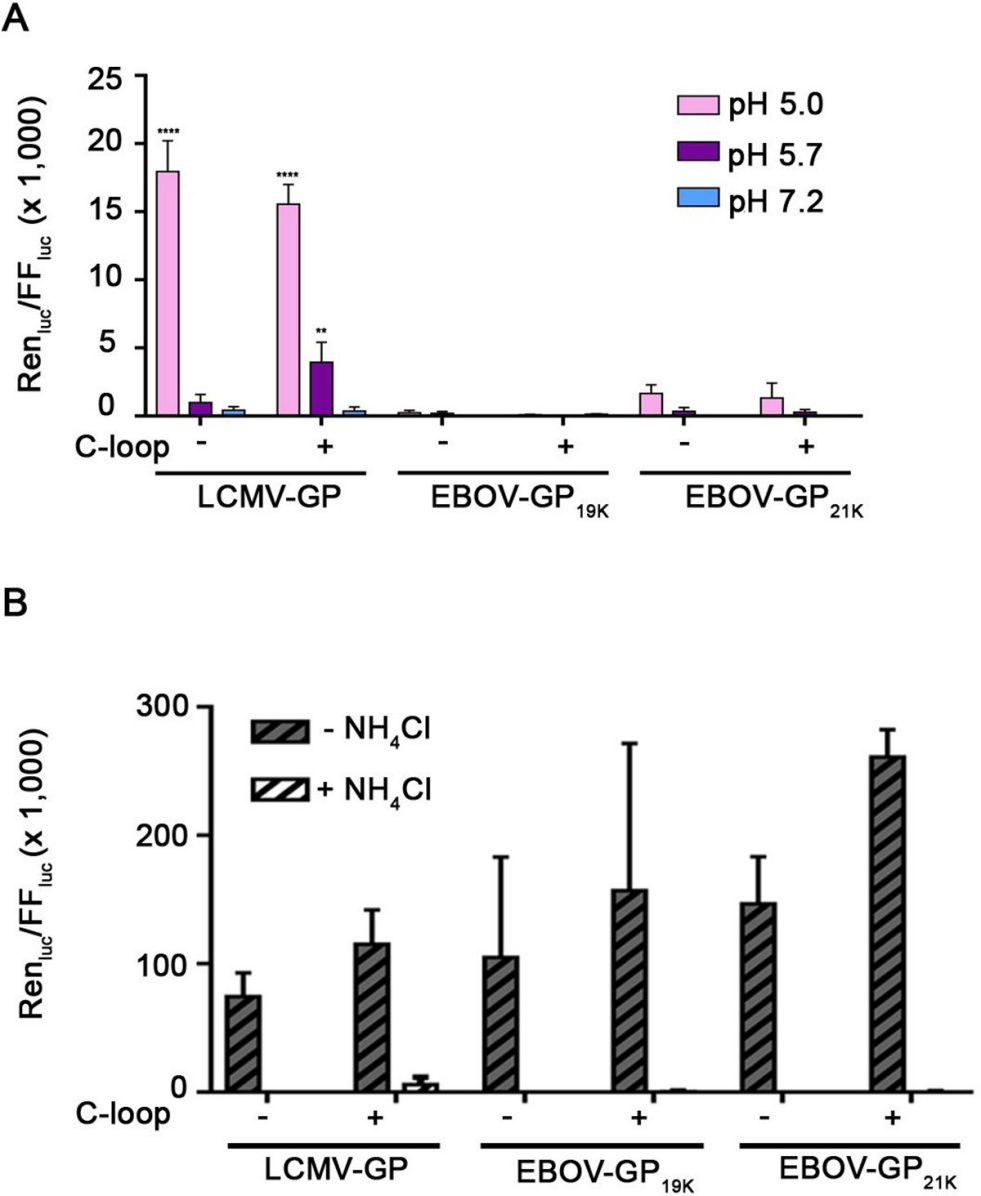
NPC1-C-loop and low pH and are not sufficient to trigger detectable forced fusion at the plasma membrane (FFPM) by infectious pseudoviruses bearing EBOV-GP_cl_. **(A):** VSV pseudoviruses bearing the indicated GP were bound at 4°C to target (COS7) cells with (+) or without (-) NPC1-C-loop at their surface that had been pre-cooled (to prevent pseudovirus internalization). The cells were then pulsed at pH 5.0, 5.7 or 7.2 for 5 min at 37°C after which time the buffer was replaced with pH 7.4 medium containing 40 mM NH_4_Cl to raise endosomal pH. (FFPM was not monitored at pH 4.5 due to excessive cell loss.) After 24 h, cells were lysed and assessed for the ratio of *Renilla* luciferase (virus entry and replication) over firefly luciferase (total cells), as described in the Methods section. **(B):** VSV pseudoviruses bearing the indicated GP (equal input as for the FFPM experiment in **A**) were added to COS7 cells (+/- C-loop, as indicated) as described above and then either mock-treated or treated with 40 mM NH_4_Cl, as indicated. The cells were then incubated at 37°C for 24 h and analyzed for Renilla divided by firefly luciferase as in **A**. Data represent averages +/- SEM from four independent experiments each performed with triplicate samples.

As a second test of the sufficiency of C-Loop and low pH for EBOV-GP_cl_ fusion, we next conducted a sensitive ‘forced fusion at the plasma membrane’ (FFPM) experiment **(Fig 2)** in which VSV pseudoviruses encoding *Renilla* luciferase and expressing GP_cl_ (**S1C Fig)**, were bound to target cells expressing NPC1 C-Loop at their surface (**S1D and S2B Figs)**. Cells with bound pseudoviruses were then briefly pulsed at pH 5.0, 5.7 or 7.2, cultured for 24 h at 37°C, and assayed for *Renilla* luciferase as a read-out of fusion, entry, and infection. Binding was conducted at 4°C to prevent pseudovirus internalization and after the pH pulse, the cells were maintained in NH_4_Cl, a lysosomotropic weak base that raises endosomal pH, to prevent fusion that might occur through the normal endocytic route. For these tests we used VSV pseudoviruses expressing 19 kDa EBOV GP, generated by thermolysin cleavage of pseudoviruses expressing full-length GP, as well as ones bearing 21 kDa EBOV GP generated with the furin site mutant described above (**S1C Fig**). As a control we showed that the infectious levels of the 19 kDa and 21 kDa EBOV GP and LCMV GP pseudoviruses used were similar, and similarly inhibited by NH_4_Cl when the pseudoviruses were allowed to enter cells via the normal endocytic route (**Fig 2B**). As seen in **Fig 2A**, whereas ample FFPM was seen for pseudoviruses bearing LCMV GP, no fusion signal above background was observed with VSV pseudoviruses bearing either form of EBOV-GP_cl_, at any pH tested.

The CCF and FFPM assays employed in **Figs 1A and 2A** monitor content mixing of reporter plasmids and the generation of a luciferase signal in the resulting fused cells. Since the findings presented thus far suggested that NPC1 C-Loop and low pH are not sufficient for EBOV-GP_cl_ to mediate complete fusion (leading to content mixing), we next asked whether they are sufficient to mediate an earlier stage, hemifusion. To do this we used the lipid mixing assay employed by Markosyan et al [26], in which effector cells expressing EBOV-GP_cl_ and labeled with the fluorescent lipid probe DiO are mixed with target cells expressing NPC1 C-loop and labeled with the fluorescent lipid probe DiI. After coculturing and exposure to different pH buffers, the cells are analyzed for hemifusion between effector and target cells by flow cytometry [26]. For our analysis the cell population that had undergone lipid mixing was distinguished from unfused cell pairs by the higher DiI intensity of (hemi)fused cells due to fluorescence resonance energy transfer between DiO and DiI, leading to an enhancement of DiI fluorescence in cells undergoing lipid mixing [32] (**S3 Fig**). As seen in **Fig 1B**, whereas a lipid mixing signal was detected with effector cells expressing LCMV GP and treated at pH 5, no hemifusion signal above background was detected for effector cells expressing EBOV-GP_cl_, whether the target cells did or did not express NPC1 C-Loop at their surface. And this is although the cells expressing EBOV-GP_cl_ showed apparently increased binding to cells that expressed NPC1 C-loop, compared to ones that did not express NPC1 C-loop, at their surface **(S3 Fig)**.

### NPC1 C-loop, low pH and additional tested factors are not sufficient to trigger detectable cell-cell fusion by GP_cl_

**Fig 3:**
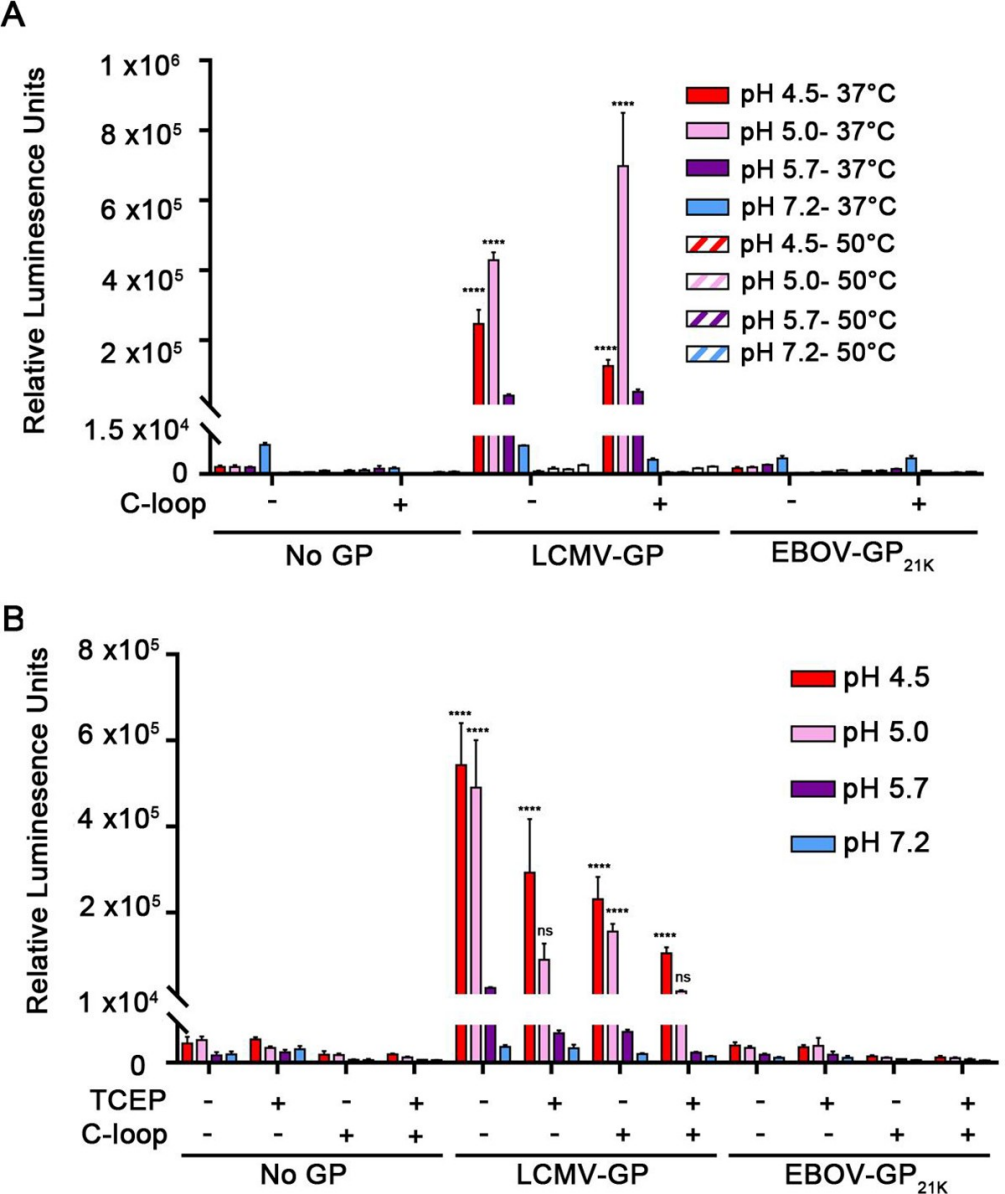
Elevated temperature and reducing conditions do not trigger detectable fusion between cells expressing EBOV-GP_cl_ and cells expressing NPC1 C-loop. **(A)** Target and effector HEK293T/17 cells were prepared, treated and analyzed for CCF, as described in the legend to **Fig 1A** except that the pH pulse was done at either 37°C or 50°C, as indicated. (**B**) Cells were prepared, treated and analyzed as in panel **A** except that, where indicated, the fusion buffer contained 5 mM TCEP. Results represent mean +/- SD of triplicate samples from one of two experiments with similar results. Statistical analyses are based on two-way ANOVA tests; each sample was compared to the respective No GP sample ****p < 0.0001.

**Fig 4:**
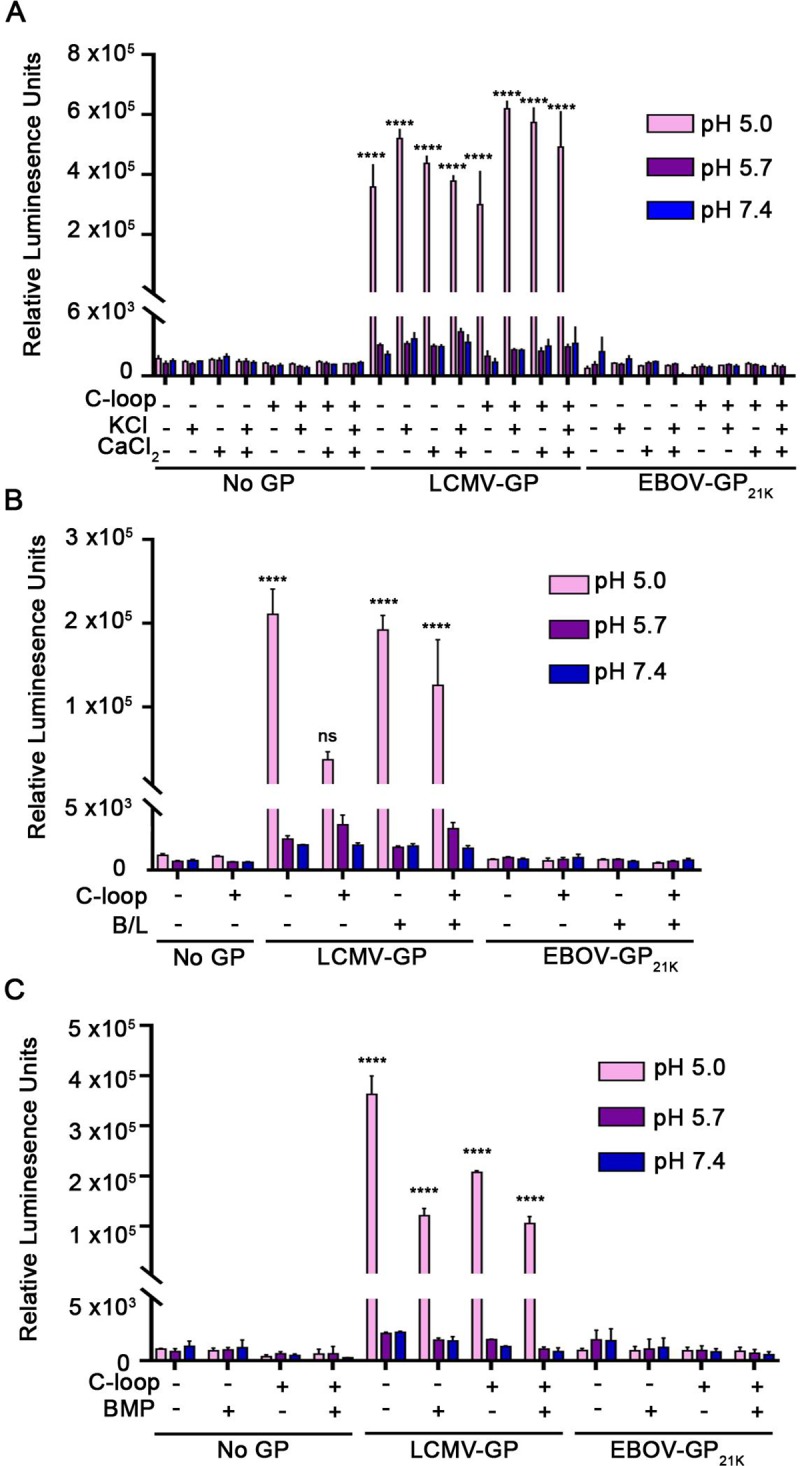
Addition of cations (Ca^++^, K^+^), cathepsins (B and L) or an anionic lipid (BMP) does not trigger detectable fusion between cells expressing EBOV-GP_cl_ and cells expressing NPC1 C-loop. Target and effector HEK293T/17 cells were prepared, treated and analyzed for CCF as described in the legend to **Fig 1A** except that the pH pulse was done, as indicated, in fusion buffer containing **(A)** 2 mM Ca^2+^ or 140 mM K^+^ or **(B)** a mixture of 5 μg/ml of pre-activated cathepsin B and 5 μg/ml of pre-activated cathepsin L (see **S5 Fig** for cathepsin activities). **(C)** Effector and target HEK293T/17 cells were prepared as described in the legend to **Fig 1A**. Cocultured cells were treated with an equimolar mixture of 5 μM BMP and delipidated BSA for 5 min at 10°C, and then subjected to a 5 min pulse at 37°C at the indicated pH. Results in each panel represent mean +/- SD of triplicate samples from one of two experiments with similar results. Statistical analyses are based on two-way ANOVA tests; samples were compared to respective No GP samples. ****p < 0.0001.

The results presented in **Figs 1 and 2** suggest that NPC1 C-Loop and low pH are not sufficient to trigger the fusion activity of EBOV-GP_cl_. We therefore tested whether five additional factors could evoke full fusion (content mixing) activity. For these tests (**Figs 3 and 4**), we employed the sensitive split luciferase CCF assay and in each experiment we included effector cells expressing LCMV GP as a positive control for CCF.

We first tested whether elevated temperature could promote fusion, since high temperature can substitute for physiological triggers that prompt conformational changes and fusion for viruses such as influenza, Sendaï, and SV5 [33–35]. Elevated temperatures also induced the EBOV GP_cl_ ectodomain to bind to liposomes at low pH [23]. However, temperatures as high as 50°C failed to induce GP_cl_ to mediate CCF to target cells expressing NPC1 C-Loop at any pH tested (**Fig 3A**). Next, we assessed the effects of the reducing agent TCEP, since reductases are present in late endosomes/lysosomes [36,37] and since 1–5 mM TCEP caused the ectodomain of EBOV-GP_cl_ to bind to liposomes, an activity diminished with the fusion loop mutant, F535R [23]. However, as seen in **Fig 3B**, no CCF above background was seen with effector cells expressing EBOV-GP_cl_ in the presence of 5 mM TCEP at any pH tested. While elevated temperature and reducing agent appear sufficient to induce the isolated GP_cl_ ectodomain to bind to liposomes [23], they are not sufficient to induce full fusion, supporting the notion that an additional factor(s) is (are) required.

The third additional factor we tested was cations, specifically Ca^++^ and K^+^. EBOV entry requires TPC2, an endosomal calcium channel [25] and the glycoproteins of Rubella [38], Severe Acute Respiratory Syndrome coronavirus (SARS-CoV) and Middle East Respiratory Syndrome coronavirus (MERS-CoV) [39,40], have been reported to require Ca^++^ for optimal functioning. K^+^ is needed for productive entry of influenza and bunyaviruses [41,42]. However, when tested using the split luciferase CCF assay, the presence or absence of either cation (at the concentrations tested) did not induce EBOV-GP_cl_ to mediate a (cell-cell) fusion signal above background at any pH tested (**Fig 4A**). A similar result was found using the FFPM assay (**S4 Fig**).

As a fourth additional factor we tested whether the addition of cathepsins B and L could evoke the fusion activity of EBOV GP_cl._ This experiment was motivated by the finding that the cysteine protease inhibitor E64d inhibits entry by 19 kDa EBOV GP [6,20]. However, as seen in **Fig 4B** application of pre-activated cathepsins B and L (**S5A Fig**) did not induce EBOV GP_cl_ to mediate CCF above background levels at any pH tested.

The last additional factor we tested was the anionic lipid Bis(Monoacylglycero)Phosphate (BMP). This lipid is enriched in late endosomes and lysosomes [43,44] and is required for fusion and entry of Dengue and Uukuniemi viruses [45,46]. However, as seen in **Fig 4C**, adding BMP did not unleash detectable fusion activity from EBOV GP_cl._

Collectively the results presented in **Figs 1–4** suggest that the C-Loop of NPC1 and low pH are not sufficient to induce EBOV-GP_cl_ to mediate detectable fusion, even when the system is supplemented with elevated temperature, a reducing agent, Ca^++^_,_ K^+^, exogenous cathepsins B and L, or the anionic lipid, BMP.

### Full length NPC1, low pH and additional factors tested are not sufficient to trigger detectable fusion by GP_cl_

The NPC1-C-loop is sufficient for binding EBOV-GP_cl_ and to promote entry and infection through endosomes [10,30]. However, other domains of NPC1 could be needed to trigger optimal EBOV GP_cl_-mediated fusion. Indeed, Miller et al. showed that while the NPC1-C-Loop could rescue entry and infection of NPC1-knock out cells, the rescue was stronger if knock-out cells were complemented with full length NPC1. They also showed, reciprocally, that while an NPC1 construct lacking the C-Loop was strongly impaired for entry and infection, constructs lacking the I or A domains were also impaired, albeit less strongly [10]. In addition, Gong et al. reported that the affinity of GP_cl_ is ~10-fold higher to full length NPC1 than to the isolated NPC1 C-loop [47].

**Fig 5:**
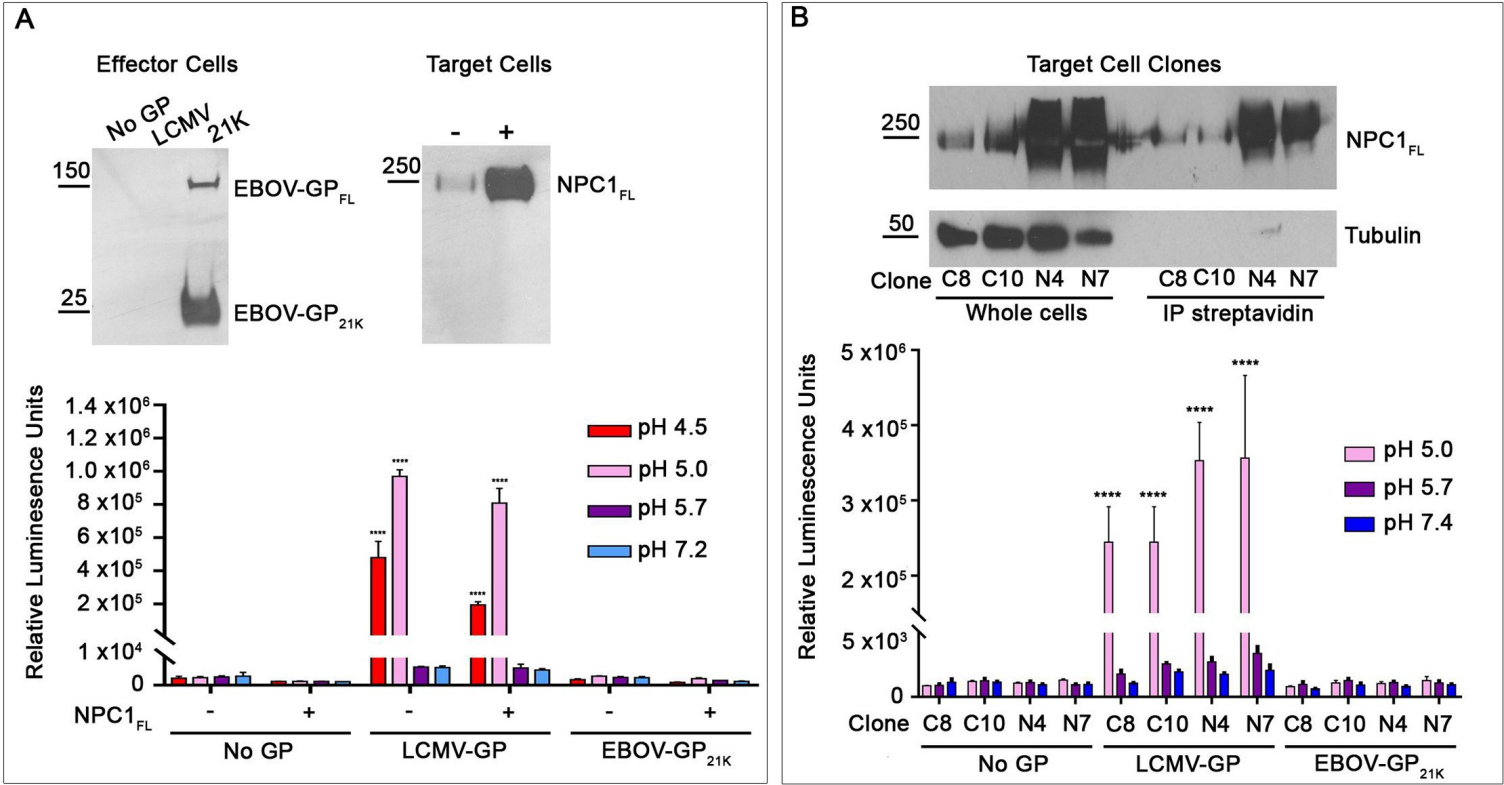
Cells expressing full length NPC1 at the cell surface do not support detectable fusion with cells expressing Ebola GP_cl_. **(A) Transient NPC1 expression:** Effector HEK293T/17 cells were transfected to express the indicated (or No) GP and DSP_1-7_. Target cells were transfected to express (+) or not (-) full-length NPC1 and DSP_8-11_. Effector and target cells were analyzed, respectively, for surface exposed EBOV-GP1 and NPC1 following surface biotinylation, avidin precipitation and blotting for EBOV GP or NPC1. Cocultures of effector and target cells were then treated for CCF and analyzed as in **Fig 1A**. **(B) Stable NPC1 expression:** (Top) Selected clones (C, control or N, expressing full-length NPC1) were analyzed for NPC1 in whole cell lysates by western blots (left set) and for cell surface exposed NPC1 as in **A**. Blotting for beta-tubulin was used as a control for protein loading (left set) and cell intactness (right set). Parallel sets of cells depicted in the gels were then used as targets in CCF experiments with effector cells expressing the indicated (or No) GP. Cocultured effector and target cells were then processed as in the graph shown in panel **A**. Results represent mean +/- SD of triplicate samples from one of two experiments with similar results. Statistical analyses are based on two-way ANOVA tests; each sample was compared to the respective No GP sample ****p < 0.0001.

We therefore asked if full-length NPC1 could induce the fusion activity of EBOV-GP_cl_. While NPC1 is mainly found in late endosomes [17,48] cells may express some endogenous NPC1 at their surface (**Fig 5A and 5B,** gels of untransfected (-) target cells and control (C) target cell clones) (also see [26]). In a first set of experiments, we transiently overexpressed NPC1 by transfecting target cells with a plasmid encoding full length NPC1 cDNA under the control of a CMV promoter. Under these conditions, a substantial amount of NPC1 was found at the cell surface, as detected by cell surface biotinylation (**Fig 5A,** gel of transfected (+) target cells). Cells transiently expressing full length NPC1 at their surface were then tested as targets in the split luciferase CCF assay with effector cells expressing EBOV-GP_cl_ at their surface (**Fig 5A,** gel of effector cells), as described above. However, as with its C-loop, full-length NPC1 did not elicit detectable content mixing CCF activity for EBOV-GP_cl_ at any pH tested, while LCMV-GP, again, induced robust CCF at low pH **(Fig 5A**). As a further test we generated stable clones of HEK293T/17 cells that express significant levels of NPC1 at their surface (**Fig 5B,** gels of clones N4, N7). Consistent with the findings with transiently transfected cells (**Fig 5A**), GP_cl_-mediated fusion was not detected with target cells that stably express full length NPC1 at their surface (**Fig 5B**).

**Fig 6:**
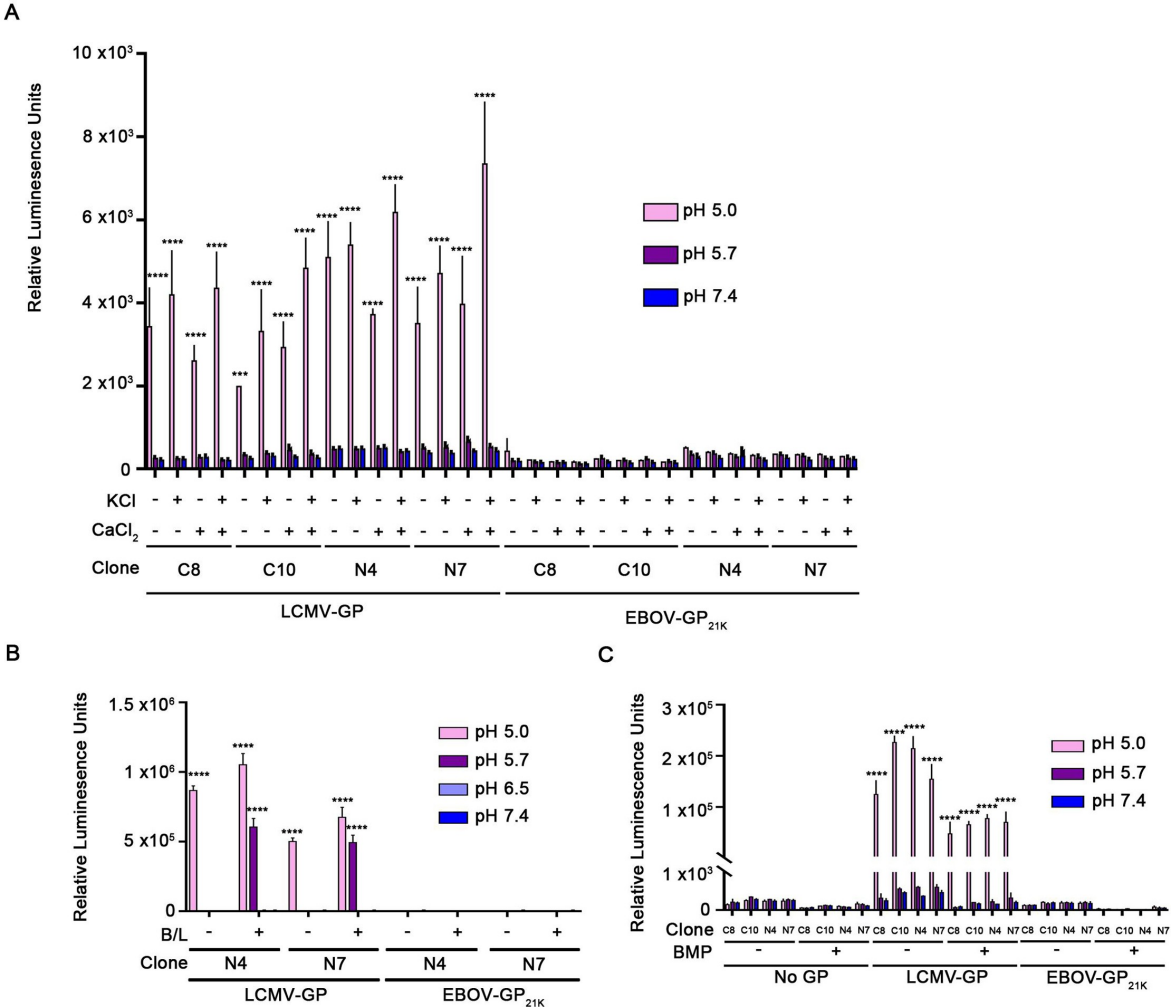
Addition of cations (Ca^++^, K^+^), cathepsins (B and L) or an anionic lipid (BMP) to stable clones expressing full length NPC1 at the cell surface does not promote detectable EBOV-GP_cl_-mediated cell-cell fusion. Effector cells expressing the indicated GP and target control or NPC1-expressing cells were prepared as described in the legend to **Fig 5B**. Cocultured cells were then treated with (**A**) Ca^++^ or K^+^, (**B**) cathepsins or (**C**) BMP as described in the legend to **Fig 3A, 3B and 3C**, respectively, except that for **Fig 6C**, the target cells were treated with an additional mixture of 5 μM BMP and 5 μM delipidated BSA for 5 min at 10°C before being cocultured with effector cells. For **Fig 6C**, after coculturing for 45 min, the cells were processed and analyzed for CCF as in **Fig 3C**. Results represent mean +/- SD of triplicate samples from one of two experiments with similar results. Statistical analyses are based on two-way ANOVA tests: for **A** and **B** each sample was compared to the pH 7.4 sample in each group and for **C** compared to the respective No GP sample. ****p < 0.0001; ***p< 0.001; **p < 0.01; *p<0.05.

Lastly, we asked whether three potentially physiologically relevant additional factors in conjunction with full length NPC1 and low pH could evoke detectable CCF activity from EBOV-GP_cl_. However, addition of the cations Ca^++^ or K^+^ (**Fig 6A**), exogenous cathepsins B and cat L (**Fig 6B and S5B Fig**), or the anionic lipid BMP (**Fig 6C**) did not promote detectable EBOV-GP_cl_-mediated CCF with target cells that stably express full length NPC1 at their surface.

## Discussion

Ebola virus (EBOV) is a highly lethal enveloped virus [1] that employs an unusually complex pathway to enter host cells [3]. After engaging attachment factors on the cell surface (which can differ on different cells), EBOV particles are engulfed by macropinocytosis and trafficked via early and late endosomes to an endocytic compartment referred to as a late endosome/lysosome (LE/Lys) [3,4,17–19,49]. *En route* the receptor binding subunit of the EBOV glycoprotein (GP) is cleaved by cathepsins B and L to generate a cleaved (~19 kDa) species [5,6] that binds to the C-Loop of Niemann-Pick-C1 (NPC1), the EBOV endosomal receptor [8–10]; fusion then ensues through acidic LE/Lys [18,19]. For most other enveloped viruses that enter cells through endosomes, even for Lassa virus, the second virus shown to use an intracellular/endosomal receptor [12,13], low pH is then sufficient to trigger conformational changes that elicit the fusion activity of the viral fusion GP [16]. The fusion GPs of SARS and MERS coronaviruses additionally require a (post receptor binding) endosomal proteolytic cleavage event [50–53].

The conditions that elicit the fusion activity of cathepsin-cleaved EBOV GP (GP_cl_) have been debated. While findings stated in Miller et al. indicated that low pH did not trigger recombinant VSV particles bearing EBOV GP_cl_ (the 19 kDa form generated by thermolysin cleavage) to fuse and thereby infect cells expressing NPC1-C-Loop at their surface [10], Markosyan et al [26] reported that NPC1 at cell surfaces is sufficient to mediate cell-cell fusion (CCF) with cells expressing EBOV GP_cl_ (see below). Because of these conflicting conclusions we re-examined the sufficiency of NPC1 to trigger the fusion activity of EBOV GP_cl._ In the process, we also assessed the potential enhancing role of additional factors shown, as outlined in the Results section, to augment fusion of other endocytosed enveloped viruses [23,33–35,38,39,45,50,53]. Our findings are consistent with and extend the result stated by Miller et al. [10]. We found that cells expressing either NPC1-C-Loop or full length NPC1 at their surface did not serve as adequate targets for detectable fusion, at any pH tested, with effector cells or pseudovirus particles bearing EBOV GP_cl_ (19 kDa or 21 kDa forms). Moreover, this apparent insufficiency was seen even if effector-target cell pairs were additionally exposed to elevated temperature, a reducing agent, Ca^++^, K^+^, the anionic lipid BMP, or cathepsins B and L.

Markosyan et al [26] monitored CCF between effector cells bearing EBOV GP_cl_ (either the 19 kDa or 21 kDa form) and target cells bearing NPC1 by scoring mixing of fluorescent lipid dyes as well as a small (622 Dalton) aqueous content dye; they also queried electrical conductivity between effector-target cell pairs exposed to pH 5.7. They concluded that very small, non-expanding fusion pores formed at both neutral and (more quickly) low pH (optimally at pH 5.7). While we employed the delivery and expression of luciferase reporter plasmids as a (different, but highly sensitive) content mixing assay, we used the same lipid mixing assay as Markosyan et al., and as per Gomez-Icazbalceta et al. [32], gated out bound (adhered) but unfused cell pairs (**S3 Fig**). With these means of analysis we did not observe a detectable lipid mixing signal with effector cells bearing EBOV GP_cl_, at any pH tested, while lipid mixing was detected with effector cells bearing LCMV GP at pH 5. Our disparate lipid mixing results could be due to technical and analytical differences. Irrespectively, the small non-expanding fusion pores reported by Markosyan et al. [26] would likely not lead to productive viral infection, being too small to pass a viral nucleocapsid. Hence, we conclude that NPC1 and low pH are not sufficient to drive the fusion activity of EBOV GP_cl_. This conclusion is consistent with x-ray analysis of the GP_cl_-NPC1-C-loop complex, indicating that only small, but likely important, conformational changes occur when EBOV GP_cl_ binds to NPC1-C-Loop at pH 5.5 (albeit as seen in crystals formed at 18°C). For example, there was no evidence that binding of NPC1 to EBOV GP_cl_ (at low pH and 18°C) causes expulsion of the fusion loop from the body of the GP_cl_ trimer [54].

Based on findings stated by Miller et al. [10] and presented in **Figs 1, 2 and 5**, we postulated that a factor(s) in addition to low pH and NPC1 is needed to elicit the fusion activity of EBOV GP_cl_. We therefore tested factors required for fusion or uncoating of other endocytosed enveloped viruses (Ca^++^, K^+^, BMP, proteases) [38,39,41,45,53], proposed for EBOV in particular (Ca^++^, additional cathepsin action, reducing agent) [6,25,55], and even the non-physiological condition of elevated temperature, which can substitute for biological viral fusion triggers [33–35]. However, none of these factors or treatments elicited detectable CCF between effector cells bearing EBOV GP_cl_ and target cells bearing NPC1 or its C-Loop (at any pH tested), while robust CCF was consistently detected with effector cells bearing LCMV GP at low pH. Although we cannot rule out that one of the tested additional factors induced hemifusion or small non-expanding fusion pores, or that CCF would be observed with a combination of these factors, we favor the hypothesis that the fusion activity of EBOV GP_cl_ requires NPC1, low pH and a yet to be identified, likely endosomal, factor. The initial observation indicating a need for an additional factor was that pseudovirus particles bearing 19 kDa EBOV GP are not infectious if target cells are pretreated with a lysosomotropic agent (which raises endosomal pH) or with the cysteine protease inhibitor E64d [6], findings that have been confirmed by others [20]. In this context it is interesting that Spence et al. [19] recently found, in live cell imaging studies employing VSV particles bearing EBOV GP_cl_, that the E64d-sensitive step follows hemifusion, i.e. is at the stage of fusion pore formation or particle uncoating. On the other hand, we have observed fusion of the isolated EBOV fusion loop [21,56] and of EBOV GP2 reconstituted into proteoliposomes [57] with liposomes at low pH, suggesting that a major role of NPC1 and the additional factor may be to move GP1 sufficiently out of the way so that GP2 can proceed along the fusion pathway [16].

There are additional caveats to our inability to detect a fusion signal for EBOV GP_cl_ under any of the conditions tested. For one, the density of NPC1 (or its C-loop) at the surfaces of the target cells employed may not have been high enough, in conjunction with low pH at 37°C, to trigger fusion. Secondly, although we tested adding the LE/Lys-abundant lipid BMP, which is needed for full fusion by Dengue and Uukuniemi viruses [45,46], the plasma membrane lipid composition may not support EBOV GP_cl_-mediated fusion, which is designed to occur in LE/Lys; the plasma membrane and LE/Lys membrane have different lipid compositions [43,44]. Or, the plasma membrane may contain a lipid or protein factor that is inhibitory to EBOV GP_cl_-mediated fusion. Another possibility is that an additional LE/Lys membrane protein, for example TPC2, is required. TPC2 is a membrane protein that mediates Ca^++^ efflux out of LE/Lys [58]; cells lacking TPC2 are strongly impaired for EBOV infection [25]. Perhaps TPC2 is needed to maintain a precise local concentration of Ca^++^ not mimicked in our experiments. Ca^++^ could be needed to stabilize the structure of the fusion loop, or for the fusion loop to interact with the LE/Lys lipid bilayer [38–40]. And lastly, another membrane protein in LE/Lys or a specific soluble component present in the lumen of LE/Lys (ion, small molecule, protein) could be required for EBOV GP_cl_ to mediate robust fusion. Indeed, multiple factors could be needed to maintain a precise ionic milieu in the endosomal lumen [59] needed to support EBOV fusion. In this context it is potentially interesting that lipid and ion transporter proteins emerged in a recent Crispr screen for host factors required for EBOV infection [60].

In conclusion, our study highlights the complexity of the EBOV fusion mechanism and adds support to the proposal that additional cues are necessary (after cleavage of GP to GPcl, binding to NPC1 and exposure to low pH) for EBOV to open, expand and sustain a robust fusion pore. Future studies are aimed at identifying the putative missing factor(s) and testing other filoviral GPs that may be more prone to fusion, as suggested in a recent report by Ruedas et al [61].

## Supporting information

S1 Fig Analysis of Ebola GP and NPC1 C-loop on cells and pseudovirus particles used for fusion experiments in Figs 1 and 2. Effector **(A)** and target (**B**) cells used for CCF experiments in **Fig 1A**. The surfaces of effector and target HEK293T/17 cells were biotinylated and analyzed for surface exposed EBOV-GP1 (**A**) or NPC1-C-loop (**B**) as described in the Methods section, blotting for, respectively. EBOV-GP and the HA epitope tag on the membrane-anchored NPC1-C-loop construct. (**C**) VSV pseudoviruses bearing EBOV-19K-GP or EBOV-21K-GP (for experiments in **Fig 2)** were analyzed by SDS-PAGE and blotting for EBOV GP. (**D**) Target cells for experiments shown in **Fig 2** were biotinylated and analyzed for surface exposed NPC1 C-loop. See Methods section for details.
(TIFF)

S2 Fig Effector cells expressing EBOV GP_CL_ and target cells expressing NPC1 C-loop are competent to bind NPC1 C-loop and EBOV GP_CL_, respectively. In Cell Westerns were performed as described in the Methods section to assess (**A**) soluble NPC1 C-loop binding to effector cells expressing EBOV GP_CL_ (21 kDa form) and (**B**) binding of VSV pseudoviruses bearing LCMV GP, EBOV GP_CL_ (19kDa form), or full-length EBOV GP to target cells expressing membrane-anchored NPC1 C-loop. Data in **A** are the averages of triplicate samples (+/- SD) from one experiment. Data in **B** are the averages from three experiments (+/- SEM), each performed with duplicate samples.
(TIFF)

S3 Fig FACS plots for experiment depicted in Fig 1B: Lipid mixing assay. Plots are for samples from one of the three experiments averaged in **Fig 1B** showing the gates imposed, as elaborated in the schematic and in the Methods section. Note that the assay measures lipid mixing, the hallmark of hemifusion. The fused (F) population (upper right section) could encompass both hemifused and fully fused cells. The bound (B) population represents cells that are adhered, but not fused. The inset Table gives the %B and %F (of all stained cells) for the indicated FACS plots.
(TIFF)

S4 Fig NPC1-C-loop, low pH and cations (Ca^++^ or K^+^) are not sufficient to trigger detectable FFPM of pseudoviruses bearing EBOV GP_cl_. **(A)** VSV pseudoviruses bearing the indicated GP were bound to pre-cooled COS7 cells, either untransfected (-) or transfected to express surface-directed NPC1-C-loop (+). After binding in the cold (to prevent internalization), cells were pulsed at the indicated pH for 5 min at 37°C in fusion buffer containing, where indicated, 2 mM Ca^++^ or 140 mM K^+^. The cells were then re-neutralized and treated with 40 mM NH_4_Cl to raise endosomal pH. After 24 h, the cells were lysed and assessed for the ratio of *Renilla* luciferase activity (virus replication) over firefly luciferase activity (number of cells). **(B)** In the same experiment, equal inputs of the pseudoviruses used in **(A)** were added to cells, either mock-treated or pre-treated with 40 mM NH_4_Cl, and incubated for 24 h at 37°C. At this time, they were analyzed for Renilla divided by firefly luciferase activity. In both panels, results are shown as means +/- SD of triplicate samples from one experiment. Statistical analyses in (**A**) are shown as the comparison of each sample with the pH 7.4 sample within each group. ****p< 0.0001 based on a two-way ANOVA test.
(TIFF)

S5 Fig Assessment of pre-activated cathepsins used for experiments depicted in Figs 4B and 6B. (**A**) Triplicate samples of pre-activated cathepsin B **(left)** and L **(right)** used for the experiment depicted in **Fig 4B** were mock-treated or treated with 1 μM CA074 or 10 μM E64d, respectively for 15 min at RT. Cathepsin substrates (see Methods section) were added and samples were incubated for 5 min at 37°C. Cathepsin activity was then measured by reading Ex. 360/Em. 460 in a Biotek plate reader. **(B)** The same analyses conducted for pre-activated cathepsin B **(left)** and L **(right)** used for the experiment depicted in **Fig 6B**. Results are shown as means +/- SD of triplicate samples from the experiments conducted in parallel with those displayed in **Figs 4B and 6B**. Statistics are based on Student’s t-test. ****p < 0.0001.
(TIFF)
