# Supplementary figures and images for: An exploration of conditions proposed to trigger the Ebola virus glycoprotein for fusion
Source: PLoS One. 2019 Jul 5;14(7):e0219312. doi: 10.1371/journal.pone.0219312 (PMC6611598; doi:10.1371/journal.pone.0219312)

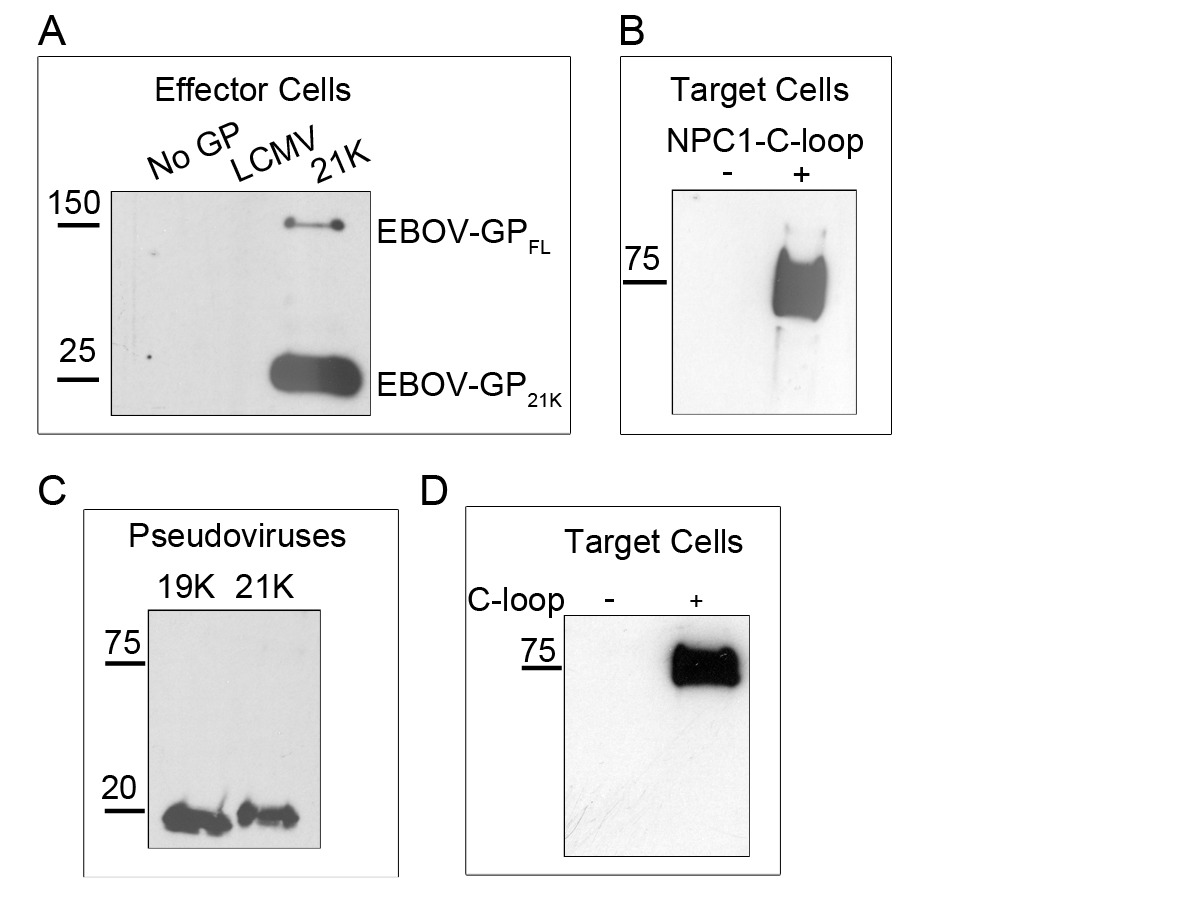

Supplement: S1 Fig — Effector (A) and target (B) cells used for CCF experiments in Fig 1A. The surfaces of effector and target HEK293T/17 cells were biotinylated and analyzed for surface exposed EBOV-GP1 (A) or NPC1-C-loop (B) as described in the Methods section, blotting for, respectively. EBOV-GP and the HA epitope tag on the membrane-anchored NPC1-C-loop construct. (C) VSV pseudoviruses bearing EBOV-19K-GP or EBOV-21K-GP (for experiments in Fig 2) were analyzed by SDS-PAGE and blotting for EBOV GP. (D) Target cells for experiments shown in Fig 2 were biotinylated and analyzed for surface exposed NPC1 C-loop. See Methods section for details. (TIFF) [file pone.0219312.s001.tiff]

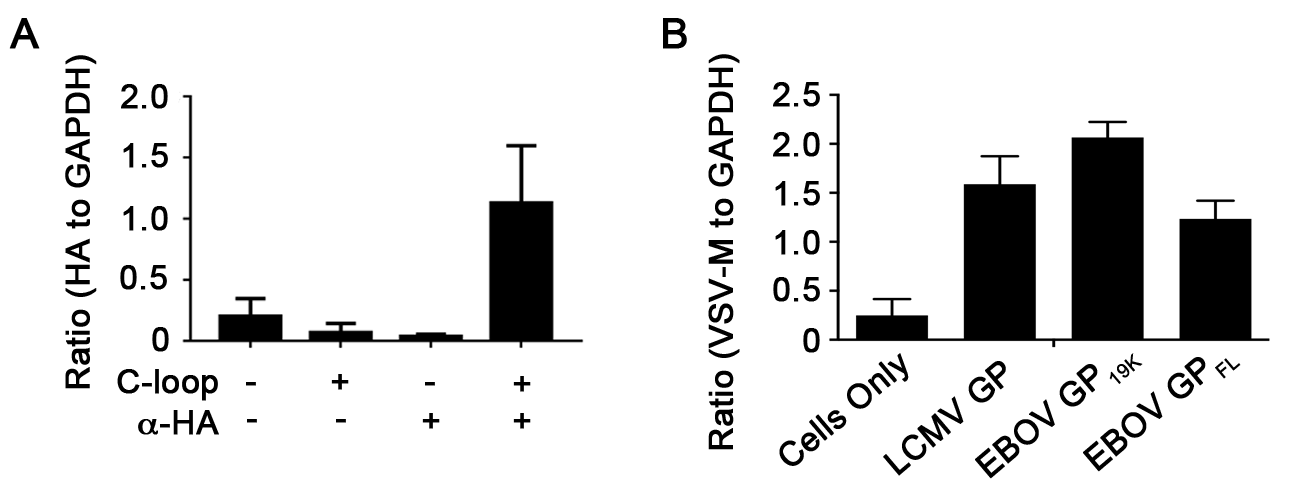

Supplement: S2 Fig — In Cell Westerns were performed as described in the Methods section to assess (A) soluble NPC1 C-loop binding to effector cells expressing EBOV GPCL (21 kDa form) and (B) binding of VSV pseudoviruses bearing LCMV GP, EBOV GPCL (19kDa form), or full-length EBOV GP to target cells expressing membrane-anchored NPC1 C-loop. Data in A are the averages of triplicate samples (+/- SD) from one experiment. Data in B are the averages from three experiments (+/- SEM), each performed with duplicate samples. (TIFF) [file pone.0219312.s002.tiff]

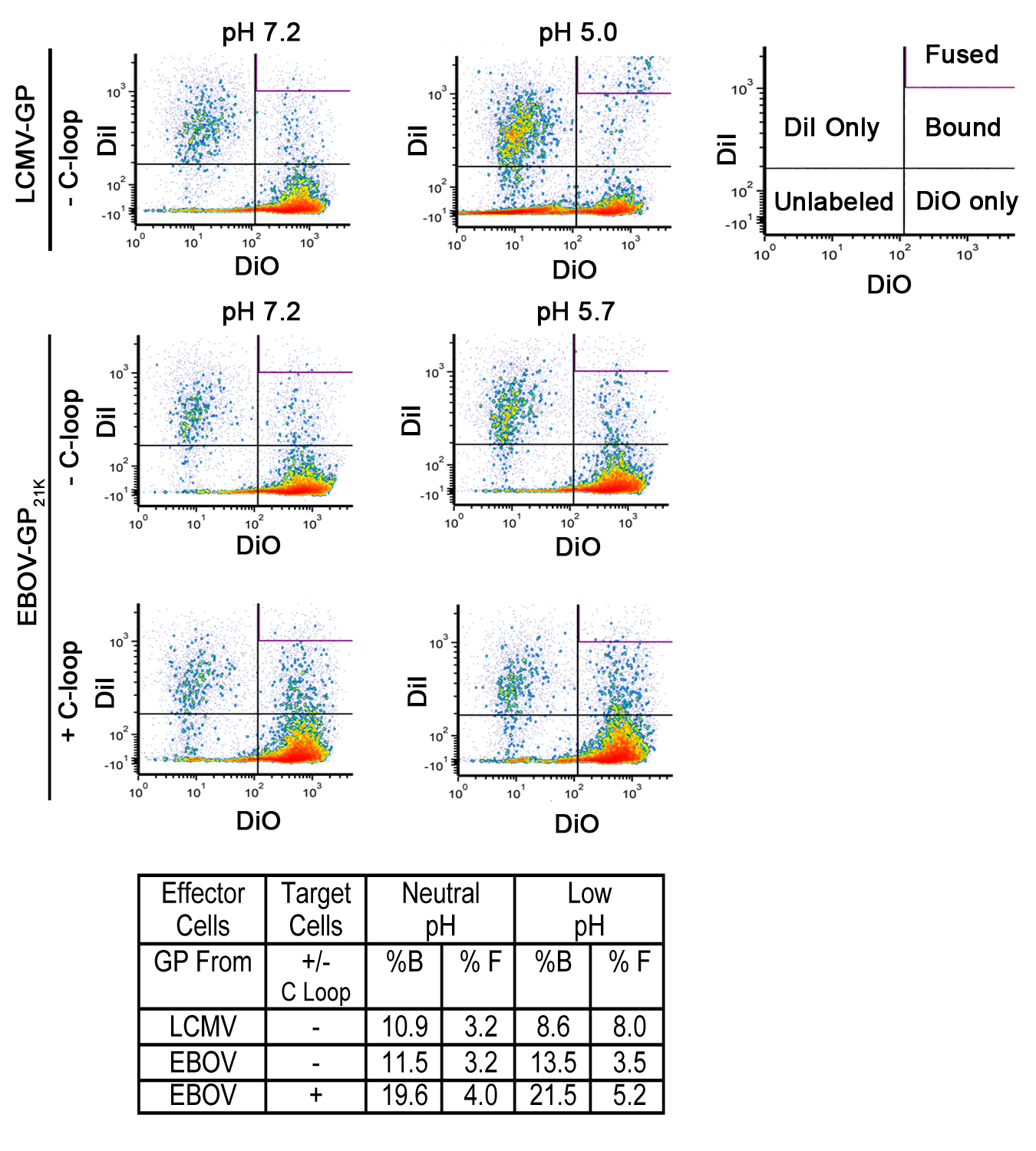

Supplement: S3 Fig — Plots are for samples from one of the three experiments averaged in Fig 1B showing the gates imposed, as elaborated in the schematic and in the Methods section. Note that the assay measures lipid mixing, the hallmark of hemifusion. The fused (F) population (upper right section) could encompass both hemifused and fully fused cells. The bound (B) population represents cells that are adhered, but not fused. The inset Table gives the %B and %F (of all stained cells) for the indicated FACS plots. (TIFF) [file pone.0219312.s003.tiff]

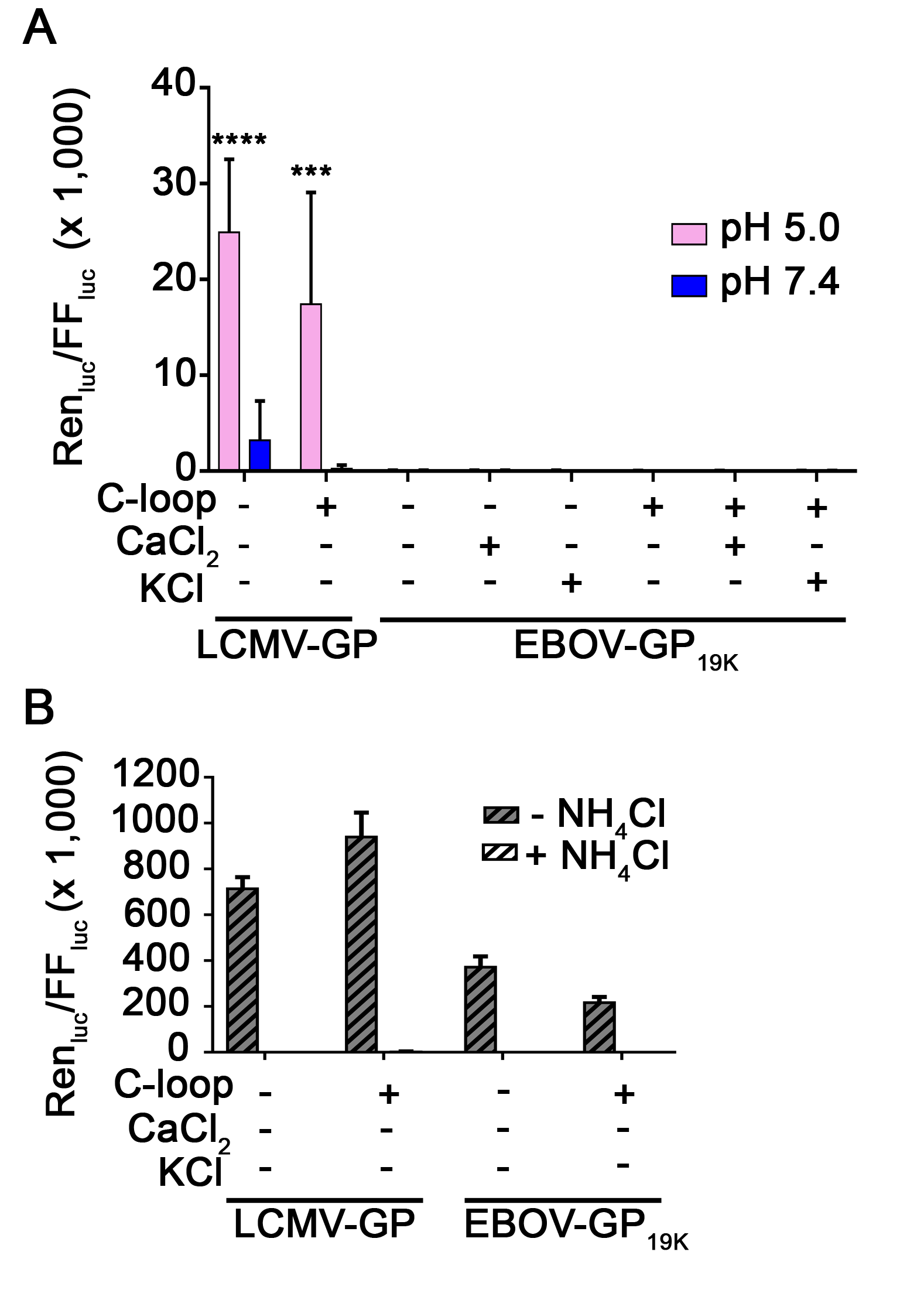

Supplement: S4 Fig — (A) VSV pseudoviruses bearing the indicated GP were bound to pre-cooled COS7 cells, either untransfected (-) or transfected to express surface-directed NPC1-C-loop (+). After binding in the cold (to prevent internalization), cells were pulsed at the indicated pH for 5 min at 37°C in fusion buffer containing, where indicated, 2 mM Ca++ or 140 mM K+. The cells were then re-neutralized and treated with 40 mM NH4Cl to raise endosomal pH. After 24 h, the cells were lysed and assessed for the ratio of Renilla luciferase activity (virus replication) over firefly luciferase activity (number of cells). (B) In the same experiment, equal inputs of the pseudoviruses used in (A) were added to cells, either mock-treated or pre-treated with 40 mM NH4Cl, and incubated for 24 h at 37°C. At this time, they were analyzed for Renilla divided by firefly luciferase activity. In both panels, results are shown as means +/- SD of triplicate samples from one experiment. Statistical analyses in (A) are shown as the comparison of each sample with the pH 7.4 sample within each group. ****p< 0.0001 based on a two-way ANOVA test. (TIFF) [file pone.0219312.s004.tiff]

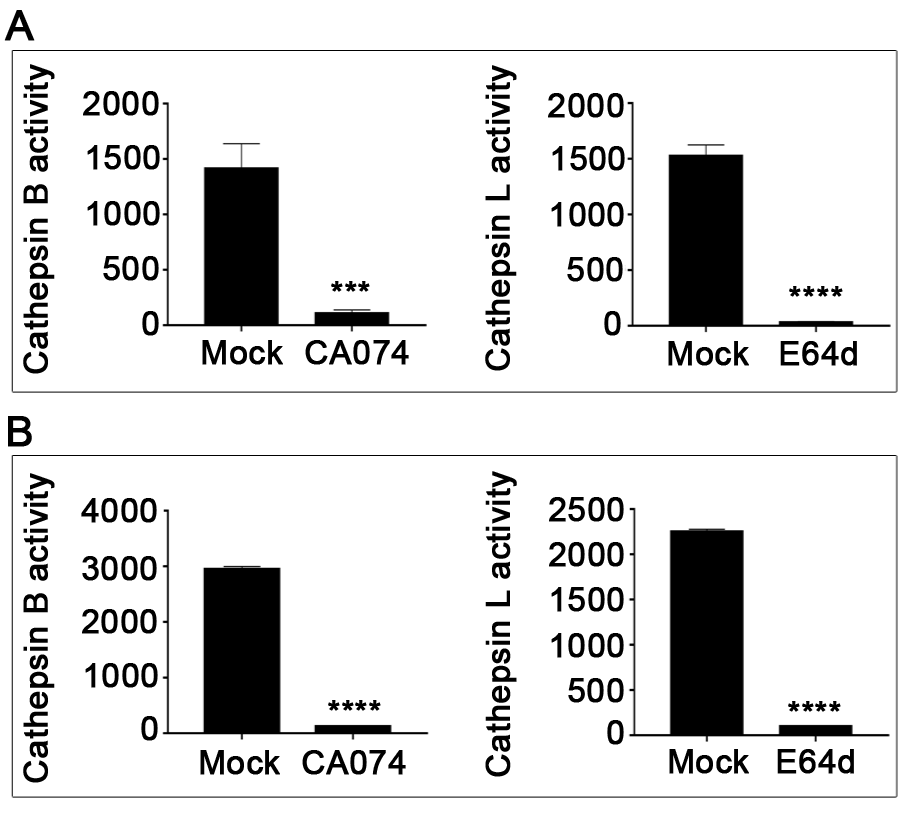

Supplement: S5 Fig — (A) Triplicate samples of pre-activated cathepsin B (left) and L (right) used for the experiment depicted in Fig 4B were mock-treated or treated with 1 μM CA074 or 10 μM E64d, respectively for 15 min at RT. Cathepsin substrates (see Methods section) were added and samples were incubated for 5 min at 37°C. Cathepsin activity was then measured by reading Ex. 360/Em. 460 in a Biotek plate reader. (B) The same analyses conducted for pre-activated cathepsin B (left) and L (right) used for the experiment depicted in Fig 6B. Results are shown as means +/- SD of triplicate samples from the experiments conducted in parallel with those displayed in Figs 4B and 6B. Statistics are based on Student’s t-test. ****p < 0.0001. (TIFF) [file pone.0219312.s005.tiff]
